# Mutational Analysis of *Aspergillus fumigatus* Volatile Oxylipins in a *Drosophila* Eclosion Assay

**DOI:** 10.3390/jof9040402

**Published:** 2023-03-24

**Authors:** Hadeel S. Almaliki, Mengyao Niu, Nancy P. Keller, Guohua Yin, Joan W. Bennett

**Affiliations:** 1 Technical Institute of Samawa, Al-Furat Al-Awsat Technical University, Samawa 66001, Iraq; 2 Department of Medical Microbiology and Immunology, University of Wisconsin-Madison, Madison, WI 53706, USA; 3 Peking University Institute of Advanced Agricultural Sciences, Shandong Laboratory of Advanced Agricultural Sciences in Weifang, Weifang 261325, China; 4 Department of Plant Biology, Rutgers, The State University of New Jersey, New Brunswick, NJ 08901, USA

**Keywords:** *Aspergillus fumigatus*, volatile organic compounds (VOCs), oxylipins, eclosion assay, *Drosophila* innate immunity

## Abstract

*Aspergillus fumigatus* is a ubiquitous opportunistic pathogen. We have previously reported that volatile organic compounds (VOCs) produced by *A. fumigatus* cause delays in metamorphosis, morphological abnormalities, and death in a *Drosophila melanogaster* eclosion model. Here, we developed *A*. *fumigatus* deletion mutants with blocked oxylipin biosynthesis pathways (∆*ppoABC*) and then exposed the third instar larvae of *D. melanogaster* to a shared atmosphere with either *A. fumigatus* wild-type or oxylipin mutant cultures for 15 days. Fly larvae exposed to VOCs from wild-type *A*. *fumigatus* strains exhibited delays in metamorphosis and toxicity, while larvae exposed to VOCs from the ∆*ppoABC* mutant displayed fewer morphogenic delays and higher eclosion rates than the controls. In general, when fungi were pre-grown at 37 °C, the effects of the VOCs they produced were more pronounced than when they were pre-grown at 25 °C. GC–MS analysis revealed that the wild-type *A. fumigatus* Af293 produced more abundant VOCs at higher concentrations than the oxylipin-deficient strain Af293∆*ppoABC* did. The major VOCs detected from wild-type Af293 and its triple mutant included isopentyl alcohol, isobutyl alcohol, 2-methylbutanal, acetoin, and 1-octen-3-ol. Unexpectedly, compared to wild-type flies, the eclosion tests yielded far fewer differences in metamorphosis or viability when flies with immune-deficient genotypes were exposed to VOCs from either wild-type or ∆*ppoABC* oxylipin mutants. In particular, the toxigenic effects of *Aspergillus* VOCs were not observed in mutant flies deficient in the Toll (*spz^6^*) pathway. These data indicate that the innate immune system of *Drosophila* mediates the toxicity of fungal volatiles, especially via the Toll pathway.

## 1. Introduction

*Aspergillus fumigatus* causes a range of diseases collectively called aspergillosis, including localized and minor infections, severe allergic bronchopulmonary aspergillosis, as well as systemic and life-threatening invasive aspergillosis [1]. Immunocompromised individuals, such as acute leukemia patients, hematopoietic cell transplant recipients, and solid-organ transplant recipients who are exposed to high concentrations of airborne *A. fumigatus* spores, are particularly vulnerable to systemic aspergillosis [2,3,4]. *A. fumigatus* grows in a wide range of temperatures with an optimal growth at 37 °C, it produces vast numbers of spores in various environments, and is a concern in modern medical facilities because of an increase in the number of its strains resistant to antifungal drugs [1,2] and the serious nature of its co-infection with COVID-19 in a condition termed CAPA (COVID-19-associated pulmonary aspergillosis) [5].

Animal models, especially invertebrate models such as *Drosophila melanogaster*, have been essential to our understanding of the virulence factors that make *A*. *fumigatus* such an effective pathogen. Wild-type *Drosophila* flies are generally resistant to microbial infections because of their robust system of innate immunity that involves several parallel signaling cascades, including the Toll pathway and the immune deficiency (Imd) pathway [6]. Consequently, Toll-deficient flies have been employed for studying *A. fumigatus* pathogenesis [7,8]. Experiments in both rodent and fly models have shown that the metabolic and developmental status of *A. fumigatus* is associated with its virulence, including the production of DHN-melanin in spores, as well as proteins and toxins that are synthesized during hyphal growth [2,9,10]. Other traits involved in fungal virulence include the ability to grow in hypoxic environments, siderophore biosynthesis, gliotoxin production, para-amino-benzoic acid metabolism and the starvation stress response [8,11,12]. Toll-deficient flies have also been used to test for *A. fumigatus* thermotolerance [13,14] and to screen for useful antifungal drugs [14].

Independently, our laboratory has developed a *D. melanogaster* eclosion bioassay using flies with a wild-type immune system for studying the physiological effects of fungal VOCs, and has successfully demonstrated the toxicity of VOCs by examining fungi grown in the laboratory after isolation from flooded homes in the aftermath of Hurricanes Katrina and Sandy [15,16]. We hypothesized that VOCs from medically important fungi might be similarly toxigenic and tested six wild-type strains of *A. fumigatus* by growing them in a shared microhabitat with the third instar larvae of *D*. *melanogaster,* such that there was no physical contact between the flies and fungi. The VOCs emitted by growing cultures of *A*. *fumigatus* and several other fungal pathogens caused significant delays in the metamorphosis and lethality of *Drosophila* flies with wild-type immune systems [11,17]. The most abundant VOCs emitted by toxigenic *A. fumigatus* included the oxylipins 1-octen-3-ol, 2-octen-1-ol, 1,3-octadeiene, 2-, cis-2-octenal, and trans-2-octenal [17].

Oxylipins are a large group of oxygenated fatty acids and their derivatives. Many oxylipins function as signaling molecules and are involved in *A. fumigatus* development and virulence [18,19,20,21]. Oxylipins are synthesized from polyunsaturated fatty acids by three major enzyme families: cyclooxygenases (COX), lipoxygenases (LOX) or P450 monooxygenases [20]. *Aspergillus* species possess both LOX enzymes and COX-like Ppo enzymes (PpoA, PpoB and PpoC) [20,22]. Several of these volatile oxylipin derivatives have been associated with fungal/insect interactions [23]. Moreover, it has been hypothesized that these VOCs contribute to “sick building syndrome”, a somewhat controversial diagnosis that is associated with human exposure to damp indoor environments [24,25,26,27].

The oxylipin linoleic acid generates volatile, eight-carbon breakdown products such as 1-octen-3-ol. Known by the common name “mushroom alcohol,” 1-octen-3-ol is characteristic of fungal metabolism [28]. Although different mold species grown on different substrates produce different combinations of VOCs, 1-octen-3-ol is usually present as part of the VOC profile [29,30,31,32,33]. Since Ppo oxylipins and the VOC 1-octen-3-ol were derived from the same substrates, it has been suspected that Ppo proteins are involved in 1-octen-3-ol metabolism; however, previous studies have yielded different conclusions on this hypothesis [34,35].

Immune-deficient flies have emerged as inexpensive models for studying invasive aspergillosis, *Aspergillus* pathogenesis, and antifungal innate immune responses [7,8,14,36]. In the present study, we continued to take advantage of the *Drosophila* model for studying the effects of fungal VOCs, using the eclosion assay larvae of both wild-type and immune-deficient flies. We applied the power of mutational analysis in two distinct ways. In the first approach, third instar *Drosophila* larvae with wild-type immune systems were exposed to VOCs from a collection of growing *A*. *fumigatus* strains, some of which carried mutations in pathways responsible for oxylipin biosynthesis. In the second approach, the VOCs generated by growing wild-type and oxylipin mutants of *A*. *fumigatus* were tested against *Drosophila* larvae that carried different immune system mutations.

We postulated that VOCs from *A. fumigatus* mutant strains with three deleted oxylipin biosynthesis genes (∆*ppoA*, ∆*ppoB*, and ∆*ppoC)* would emit fewer VOCs, at lower concentrations, and have less negative impact on metamorphosis compared to exposure to VOCs from the wild-type *A. fumigatus*. We then confirmed this hypothesis using GC–MS analysis to determine the volatile profile of wild-type Af293 and its triple mutant strain. We also tested the effects of VOCs from the different *Aspergillus* strains against the *Drosophila* larvae that carry mutant blocks in three different aspects of the fly innate immune system: nitric oxide synthase, and the Toll and Imd pathways. Because the *Drosophila* eclosion test we had developed for assaying the toxicity of fungal VOCs used flies with a wild-type immune system, we postulated that by repeating the *Drosophila* eclosion test with flies carrying defects in their innate immune response (like those used in the *Drosophila* model for aspergillosis)*,* flies would show either the same response or an even greater sensitivity to the VOCs released by *A. fumigatus* cultures. Unexpectedly, the immune-deficient flies were more resistant to the toxigenic effects of *Aspergillus* VOCs than the flies with a wild-type immune system were.

## 2. Materials and Methods

### 2.1. Fungal Strains and Media

The strain numbers, genotypes and literature citations for *A. fumigatus* strains are listed in Table 1. Stock cultures were maintained on potato dextrose agar (PDA, Difco Laboratories, Detroit, MI, USA) or on *Aspergillus* glucose minimal medium (GMM). For all *Drosophila* exposure experiments, the fungi were grown on 25 mL of PDA in 6 oz. *Drosophila* stock bottles (Genesee Scientific, San Diego, CA, USA) prior to placing the stock bottles into a common microcosm with fly larvae (see reference [17] for a diagram showing the exposure system).

The optimal growth temperature for *A. fumigatus* is 37 °C, and this is the temperature at which these fungi grow as human pathogens. However, *Drosophila* flies do not grow at this temperature. Therefore, in some experiments, fungi were pre-grown for 3 days at 37 °C and then affixed to the bottles containing fly larvae where the ensuing 15-day exposure experiment was conducted at 25 °C. Parallel experiments were conducted with fungi pre-grown at 25 °C for 5 days in order to determine whether the fungal growth temperature was associated with different VOC profiles and the associated toxicological effects.

### 2.2. Aspergillus fumigatus Mutant Construction

Fungal mutants with simultaneous deletions of *ppoA*, *ppoB*, and *ppoC* genes were created in two *A. fumigatus* genetic backgrounds, Af293 and CEA17∆*ku80*, to yield Af293∆*ppoABC* and CEA17∆*ku80*∆*ppoABC*. Briefly, to create the Af293 triple mutant, a previously published Af293∆*ppoC pyrG1* strain, TDWC3.4 [44], was used as the parental strain in which *ppoA* and *ppoB* were subsequently deleted, using the selectable markers *A*. *parasiticus pyrG* and *hph*. To create triple deletion strain in the CEA17∆*ku80* background, a CEA17∆*ku80*∆*argB*∆*ppoA* strain was first created from the TMN2.1 (CEA17∆*ku80*∆*argB*∆*pyrG*) parental strain, using *A. parasiticus pyrG* as the selection marker [45]. The resultant intermediate strain was subsequently deleted for *ppoC* and *ppoB* using *A. fumigatus argB* and *hph* selectable markers to generate CEA17∆*ku80* ∆*ppoABC*. The physiological tests for fungal strains are shown in supplementary Figure S2.

All the primers used for strain construction are listed in Supplementary Table S1. DNA transformation constructs were created through double-joint PCR using a published protocol [46]. Protoplast generation and transformation were performed according to the previously published protocol [47]. The *ppoA* deletion construct was amplified from pDWC4.2 (GF ppoA del Cassette F and GF ppoA del Cassette R) and used to transform TDWC3.4 and TMN2.1, resulting in the prototroph Af293∆*ppoC*∆*ppoA* (TMN20.11) and the arginine auxotroph CEA17∆*ku80*∆*argB*∆*ppoA* (TMN19.1). A deletion cassette for *ppoC* (*AFUB_037060*) was constructed by fusing the ~1 kb 5’ and 3’ flanking regions of the gene with *A. fumigatus argB*, amplified from pJMP4 (∆*ppoC::A.f. argB* cassette). A deletion cassette for *ppoB* (*AFUA_4g00180* for Af293 or *AFUB_100690* for CEA10) was constructed by fusing the ~1 kb 5’ and 3’ flanking regions of the gene with the recyclable hygromycin B resistance gene *hph* from pSK529 (gift from Dr. Sven Krappmann) [48]. Transformants were subsequently grown on minimal medium with 0.1% xylose to recycle the hygromycin B marker. Deletion of *ppoC* in TMN19.1 yielded a prototrophic strain CEA17∆*ku80*∆*ppoA*∆*ppoC* (TMN28.2). Lastly, *ppoB* was deleted in TMN20.11 and TMN28.2 to create Af293∆*ppoABC* (TMN31.10) and CEA17∆*ku80*∆*ppoA*BC (TMN 32.1). All transformants were first screened through PCR in order to incorporate the construct and the absence of the *ppo* gene. Southern blotting, followed by the hybridization of αP^32^-dCTP-labeled 5’ and 3’ flank regions, were used to confirm transformants with single integration (Supplementary Figure S1).

### 2.3. Drosophila melanogaster Strains and Eclosion Assay

Wild-type and immune-deficient mutant *Drosophila* strains were obtained from the Bloomington *Drosophila* Stock Center (BDSC) at Indiana University (Bloomington, IN, USA). The BDSC ID numbers, phenotypes, genotypes, human ortholog and literature citations for these strains are given in Table 1.

The *NOS* and *Rel^E20^* (Imd) mutants carried the white-eyed genotype. The double mutant flies *Rel^E20^spz^4^* (Imd and Toll) carried the red-eyed genotype. Therefore, controls for the respective exposure experiments consisted of white-eyed or red-eyed flies carrying intact immune systems. All *Drosophila* stocks were maintained in Ward’s Instant *Drosophila* medium (WARD’s Natural Science, Rochester, NY, USA). The assay was performed according to our previous studies [17]. Briefly, a *Drosophila* bottle with pre-grown *Aspergillus* cultures was taped to a Petri plate cover that had been punctured to create an opening, and then paired with a Petri plate bottom containing larval growth medium and 15 third instar larvae. Exposure to the emitted fungal VOCs continued during the ensuing stages of metamorphosis. For experiments with *Aspergillus* mutants, control larvae were exposed to PDA medium without any fungi. For experiments with *Drosophila* carrying mutations in their innate immune system, the control larvae were red-eyed or white-eyed flies with wild-type immune systems. After sealing the fungal cultures and larvae together, the bottle-plate microhabitats were incubated at 25 °C, with rotation at 50 rpm for 15 days. Each day, the numbers of larvae, pupae, and adult flies were counted.

### 2.4. Gas Chromatography–Mass Spectrometry (GC–MS) Analyses of VOCs Emitted by Wild-Type and the Triple Ppo Mutant of A. fumigatus

The wild-type *A*. *fumigatus* Af293 and the triple *ppo* mutant Af293∆*ppoABC* (TMN31.10) strains were grown on PDA in 250 mL flasks at 25 °C for 5 days or 37 °C for 3 days. Sterile PDA media and an air blank were used as the negative controls. VOC sampling and analysis were conducted as described previously [17]. The samples were purged with air at 100 mL/min at room temperature for one hour. Volatile and semi-volatile outgas products were trapped onto Tenax (modified poly[phenylene]oxide) traps. The Tenax traps were spiked with 1.0 µg of benzene-d6, toluene-d8, and naphthalene-d8 as the internal standards and purged with nitrogen for 90 min at 50 mL/min to remove water from the traps. Compounds were identified by comparing the spectra obtained from the *Aspergillus* samples with those from a reference library (NIST 08 Mass Spectra Library, Gaithersburg, MD, USA). Independent replicates were performed for each strain.

### 2.5. Statistical Analysis

The student *t*-test was used to determine the significant differences in the number of flies at each metamorphic stage with exposure to VOCs, which were emitted from *Aspergillus* wild-type and triple mutant strains. In total, 15 third instar larvae were used for each treatment, three replicates were performed for each strain, and the experiment was repeated twice. The *t*-tests for the number of fly larvae, pupae, and adults were calculated in Excel on the 4th, 8th, and 15th days, respectively.

## 3. Results

### 3.1. Aspergillus fumigatus Triple Mutant Construction

*Aspergillus fumigatus* Af293 and CEA17∆*ku80* strains were used to create Af293∆*ppoABC* and CEA17∆*ku80*∆*ppoABC* mutants with simultaneous deletions of *ppoA*, *ppoB*, and *ppoC* genes. To validate the constructs, PCR was used to screen all transformants and to verify the absence of the *ppo* genes. Then, Southern analyses were performed to further confirm these mutants for single integration (Supplementary Figure S1).

### 3.2. Effects of Aspergillus VOCs on the Metamorphic Stages of the White-Eyed w^1118^, Nitric Acid Synthase Mutation (NOS), and the Imd Pathway Drosophila mutant (Rel^E20^)

**Figure 1:**
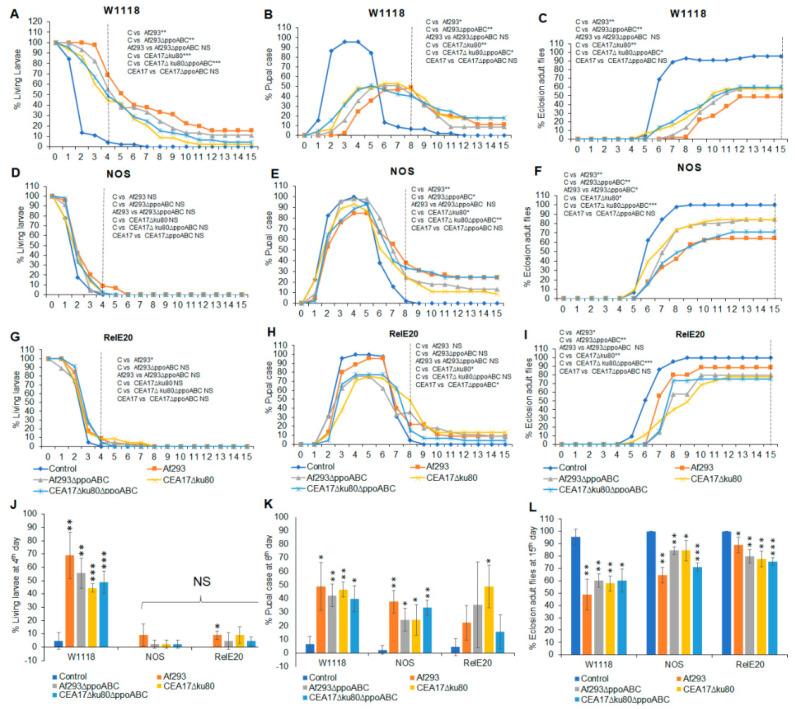
Effects of VOCs emitted from four different *A*. *fumigatus* strains pre−grown on PDA at 25 °C for 5 days on the third instar white−eyed fly larvae over 15 days of continuous exposure. The percentages of living larvae, pupal case, and eclosion adult flies are as follows: *w^1118^* living larvae (**A**), *NOS* larvae (**D**), and *Rel^E20^* larvae (**G**); *w^1118^* pupal case (**B**), *NOS* pupal case (**E**), and *Rel^E20^* pupal case (**H**); and *w^1118^* eclosion adult flies (**C**), *NOS* eclosion adult flies (**F**), and *Rel^E20^* eclosion adult flies (**I**). The statistical analyses were performed for the living larvae on the 4th day (**J**), the pupal case on the 8th day (**K**), and the eclosion adult flies on the 15th day (**L**). A dashed vertical line denotes the days for statistical analysis (N = 180). * Represents the significant differences between different treatments, where 0.005 < * *p* < 0.05, 0.0005 < ** *p* < 0.005, *** *p* < 0.0005; NS, *p* > 0.05.

**Figure 2:**
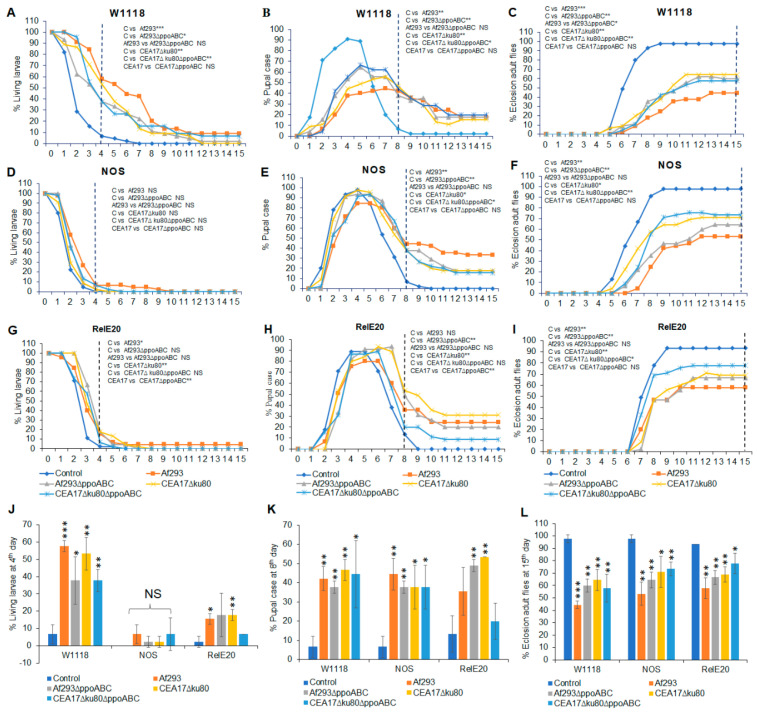
Effects of VOCs emitted from four different *A*. *fumigatus* strains pre−grown on PDA at 37 °C for 3 days on the third instar white−eyed fly larvae over 15 days of continuous exposure. The percentages of living larvae, pupal case, and eclosion adult flies are as follows: *w^1118^* living larvae (**A**), *NOS* larvae (**D**), and *Rel^E20^* larvae (**G**); *w^1118^* pupal case (**B**), *NOS* pupal case (**E**), and *Rel^E20^* pupal case (**H**); and *w^1118^* eclosion adult flies (**C**), *NOS* eclosion adult flies (**F**), and *Rel^E20^* eclosion adult flies (**I**). The statistical analyses were performed for the living larvae on the 4th day (**J**), the pupal case on the 8th day (**K**), and the eclosion adult flies on the 15th day (**L**). A dashed vertical line denotes the days for statistical analysis (N = 180). * Represents the significant differences between different treatments, where 0.005 < * *p* < 0.05, 0.0005 < ** *p* < 0.005, *** *p* < 0.0005; NS, *p* > 0.05.

**Figure 3:**
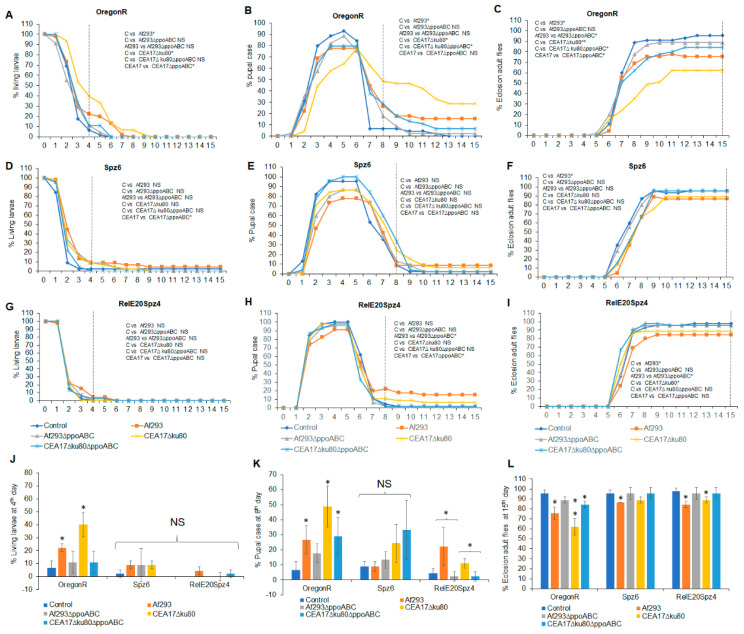
Effects of VOCs emitted from four different *A*. *fumigatus* strains pre−grown on PDA at 25 °C for 5 days on the third instar red−eyed fly larvae over 15 days of continuous exposure. The percentages of living larvae, pupal case, and eclosion adult flies are as follows: *Oregon^R^* living larvae (**A**), *Spz^6^* larvae (**D**), and *Rel^E20^Spz^4^* larvae (**G**); *Oregon^R^* pupal case (**B**), *Spz^6^* pupal case (**E**), and *Rel^E20^Spz^4^* pupal case (**H**); and *Oregon^R^* eclosion adult flies (**C**), *Spz^6^* eclosion adult flies (**F**), and *Rel^E20^Spz^4^*eclosion adult flies (**I**). The statistical analyses were performed for the living larvae on the 4th day (**J**), the pupal case on the 8th day (**K**), and the eclosion adult flies on the 15th day (**L**). A dashed vertical line denotes the days for statistical analysis (N = 180). * Represents the significant differences between different treatments, where 0.005 < * *p* < 0.05, 0.0005 < ** *p* < 0.005; NS, *p* > 0.05.

**Figure 4:**
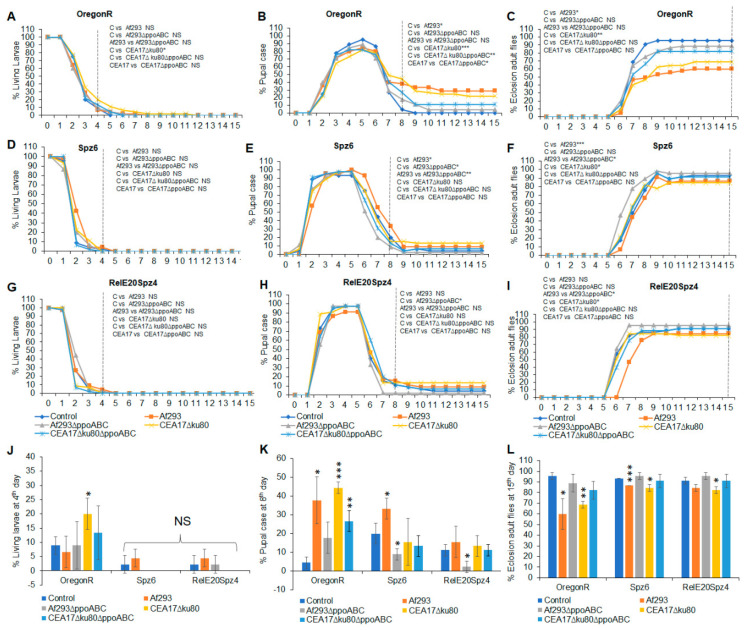
Effects of VOCs emitted from four different *A*. *fumigatus* strains pre−grown on PDA at 37 °C for 3 days on the third instar red−eye fly larvae over 15 days of continuous exposure. The percentages of living larvae, pupal case, and eclosion adult flies are as follows: *Oregon^R^* living larvae (**A**), *Spz^6^* larvae (**D**), and *Rel^E20^Spz^4^* larvae (**G**); *Oregon^R^* pupal case (**B**), *Spz^6^* pupal case (**E**), and *Rel^E20^Spz^4^* pupal case (**H**); and *Oregon^R^* eclosion adult flies (**C**), *Spz^6^* eclosion adult flies (**F**), and *Rel^E20^Spz^4^* eclosion adult flies (**I**). The statistical analyses are performed for the living larvae on the 4th day (**J**), the pupal case on the 8th day (**K**), and the eclosion adult flies on the 15th day (**L**). A dashed vertical line denotes the days for statistical analysis (N = 180). * Represents the significant differences between different treatments, where 0.005 < * *p* < 0.05, 0.0005 < ** *p* < 0.005, *** *p* < 0.0005; NS, *p* > 0.05.

Using the *Drosophila* eclosion bioassay, the number of flies at each metamorphic stage were counted. White-eyed wild-type or immune-deficient *Drosophila* mutants were grown in a shared atmosphere, with VOCs emitted by one of the *A*. *fumigatus* wild-type strains (Af293 or CEA17∆*ku80*) or their respective triple *ppo* mutant strains (Af293∆*ppoABC* or CEA17∆*ku80*∆*ppoABC*). For each set of experiments, *A. fumigatus* strains were pre-grown at either 25 °C (Figure 1 and Figure 3) or 37 °C (Figure 2 and Figure 4), prior to being placed in a shared atmosphere with the *Drosophila* third instar larvae for 15 days at 25 °C.

The number of flies at each stage of metamorphosis in the wild-type and immune-deficient white-eyed *Drosophila* flies, after exposure to a common atmosphere with VOCs emitted by wild-type and *ppo* mutants of *A*. *fumigatus,* are shown in Figure 1 and Figure 2. In Figure 1A–C and Figure 2A–C, the effects of fungal VOCs are tested on wild-type *w^1118^* flies; in Figure 1D–F and Figure 2D–F, they are tested on nitric acid synthase (*NOS*) mutant flies; and in Figure 1G–I and Figure 2G–I, they are tested on Imd pathway (*Rel^E20^*) mutant flies.

All white-eyed flies exposed to VOCs from both wild-type and mutant A. *fumigatus* grown at 25 °C (Figure 1) or 37 °C (Figure 2) showed significant delays in all stages of morphogenesis. After the eight days, almost all control flies had eclosed into adults (Figure 1C and Figure 2C), while most of the flies exposed to VOCs from wild-type *A. fumigatus* VOCs had never pupated or remained as pupae. With one exception, as hypothesized, flies exposed to VOCs from *A. fumigatus* strains with deleted *ppo* pathway genes were more likely to metamorphosize into adults than flies exposed to VOCs from wild-type *A. fumigatus*. This exception was for flies carrying the *Rel^E20^* mutation, for which exposure to VOCs from wild-type *Aspergillus* pre-grown at 25 °C was less toxic than exposure to VOCs from *Aspergillus ppo* mutants (Figure 1I). Moreover, when pre-grown at 37 °C, the VOCs emitted by wild-type *A. fumigatus* Af293 were associated with lower eclosion rates for strain *Rel^E20^* than for those exposed to VOCs emitted by the *ppo* mutants (Figure 2I). Unexpectedly, the most significant differences in eclosion numbers we observed were not between exposures to VOCs from the wild-type *Aspergillus* and its *ppo* mutants, or for the *Aspergillus* strains pre-grown at different temperature (25 °C or 37 °C), but rather for the *Drosophila* strains used in the eclosion test.

The wild-type *W^1118^* flies were negatively affected by VOCs emitted by both Af293 and its ∆*ppoABC* triple mutant. However, the white-eyed *Drosophila* strain, carrying a blocked mutation in the Imd pathway (*Rel^E20^*), was more resistant to the toxic effects of VOCs emitted by Af293 than the white-eyed *W^1118^* strain with an intact immune system (Figure 1I, *p* < 0.05). After 15 days of continuous exposure to VOCs at 25 °C, 49% of the *Drosophila w^1118^* flies (i.e.*,* those with an intact immune system) had eclosed into adults (Figure 1C,L), while 64% of flies carrying the heterozygous *NOS* mutation had eclosed (Figure 1F,L) and 89% of the *Rel^E20^* strain flies had eclosed (Figure 1I,L). The respective figures for fungi pre-grown at 37 °C were 44% for *w^1118^ (*Figure 2C,L), 53% for *NOS* (Figure 2F,L), and 57% for *Rel^E20^* (Figure 2I,L). In summary, at both temperatures tested, fly strains carrying a mutation in the Imd pathway (*Rel^E20^*) were less susceptible to the toxic effects of VOCs produced by the four *A*. *fumigatus* strains than the wild-type flies or flies carrying the *NOS* mutation (Figure 1L and Figure 2L).

### 3.3. Effects of VOCs Emitted by Wild-Type and Mutant A. fumigatus on the Metamorphic Stages of the Red-Eyed Oregon^R^, Toll Pathway Mutant (spz^6^), and Imd and Toll Pathway Drosophila Mutant (Rel^E20^spz^4^)

Compared to white-eyed *Drosophila* flies, the red-eyed Oregon^R^ strain showed fewer differences in terms of exposure to the VOCs from fungi pre-grown at different temperatures. With few exceptions, similar numbers of flies were observed at each metamorphic stage when exposed to VOCs from the *A*. *fumigatus* strains pre-cultured at 25 °C or 37 °C (Figure 3 and Figure 4). When the flies were exposed to VOCs from the wild-type *A*. *fumigatus* Af293 strain pre-grown at either temperature, 62–76% of the larvae eclosed into adults after 15 days. In contrast, when larvae were exposed to VOCs from the *A*. *fumigatu*s strains in which the three *ppo* genes were absent (Af293∆*ppoABC* or CEA17∆*ku80*∆*PPOABC*), toxicity was significantly reduced and 88–89% of larvae eclosed into adults after 15 days at 25 °C or 37 °C (Figure 3C,L and Figure 4C,L). Moreover, in all cases, fewer delays in metamorphosis were observed when immune-deficient flies were exposed to VOCs emitted by any of the tested *Aspergillus* strains. Eclosion at 15 days ranged from 82% for larvae exposed to VOCs from *A*. *fumigatus* CEA17∆*ku80* pre-grown at 25 °C (Figure 3L), to 96% for larvae exposed to VOCs from its triple mutant CEA17∆*ku80*∆*ppoABC* pre-grown at 37 °C (Figure 4L). The larvae carrying the double mutation in the Imd and Toll fly immune pathways (*Rel^E20^Spz^4^*) were more resistant to the toxicity of fungal VOCs than those of the wild-type *Oregon^R^* strain (Figure 3L and Figure 4L).

### 3.4. Comparison of Eclosion Rates of the White-Eyed and Red-Eyed Drosophila Flies Exposed to VOCs from Wild-Type and A. fumigatus Mutants

**Figure 5:**
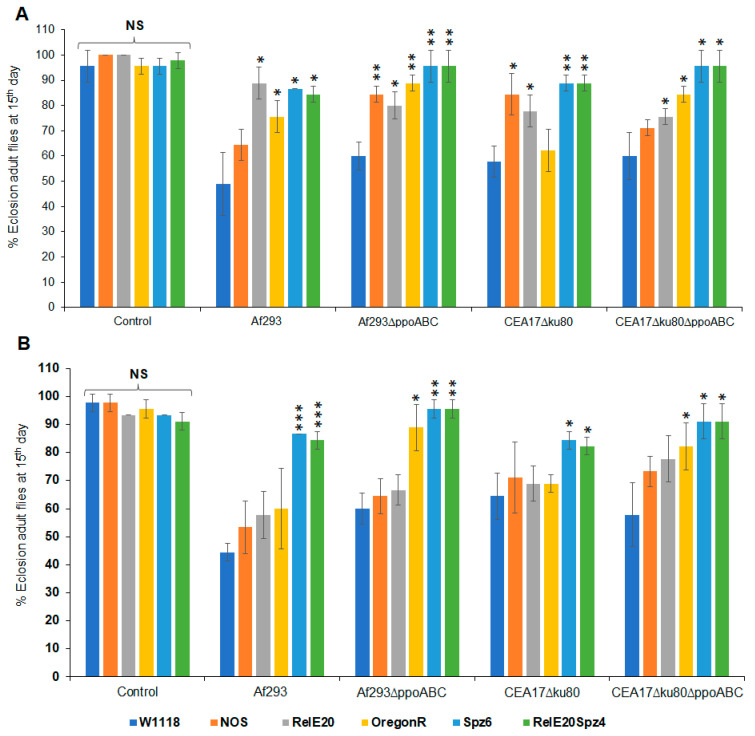
Comparative analyses of the white−eyed and red−eyed *Drosophila* male adults exposed to VOCs emitted from four different *A. fumigatus* strains. Percentages of eclosion adult flies exposed to VOCs emitted by Af293, Af293∆*ppoABC*, CEA17∆*ku80*, and CEA17∆*ku80*∆*ppoABC* pre−grown at 25 °C for 5 days are shown in (**A**); at 37 °C for 3 days are shown in (**B**). * Represents the significant differences between different treatments, where 0.005 < * *p* < 0.05, 0.0005 < ** *p* < 0.005, *** *p* < 0.0005; NS, *p* > 0.05.

The eclosion rates of the six *Drosophila* strains after 15 days of continuous exposure to VOCs emitted by wild-type or the *A*. *fumigatus* oxylipin mutants are shown in Figure 5. The highest eclosion rates were exhibited by larvae grown in a common atmosphere with the VOCs emitted by the triple oxylipin mutants (Af293∆*ppoABC* or CEA17∆*ku80*∆*ppoABC*). In general, larvae from the red-eyed flies were more resistant to fungal VOCs than those of white-eyed flies. When focusing just on the flies carrying the white-eyed genotype, larvae that carried mutations in the *NOS* or Imd immune pathways (*Rel^E20^*) were more resistant to the toxigenic effects of fungal VOCs than those of the white-eyed *w^1118^* flies with a wild-type immune system. When focusing just on flies carrying the red-eyed genotype, flies carrying the Toll mutation *Spz^6^* or a double mutation *Rel^E20^Spz^4^* in both the Imd and Toll pathway showed the fewest changes in metamorphosis or eclosion. For example, after 15 days of exposure to VOCs emitted by the *A*. *fumigatus* Af293 strain that had been pre-grown at 25 °C for 5 days, only 60 % of the wild-type Af293 flies had eclosed into adults; however, the eclosion rates for *Spz^6^* and *Rel^E20^Spz^4^* were 87% and 84%, respectively (Figure 5A). Similar results were observed for flies that were exposed to VOCs emitted by the *A*. *fumigatus* Af293 strain that had been pre-grown at 37 °C for 3 days (Figure 5B).

### 3.5. GC–MS Analyses of A. fumigatus Wild-Type Af293 and Its Triple Mutant

**Figure 6:**
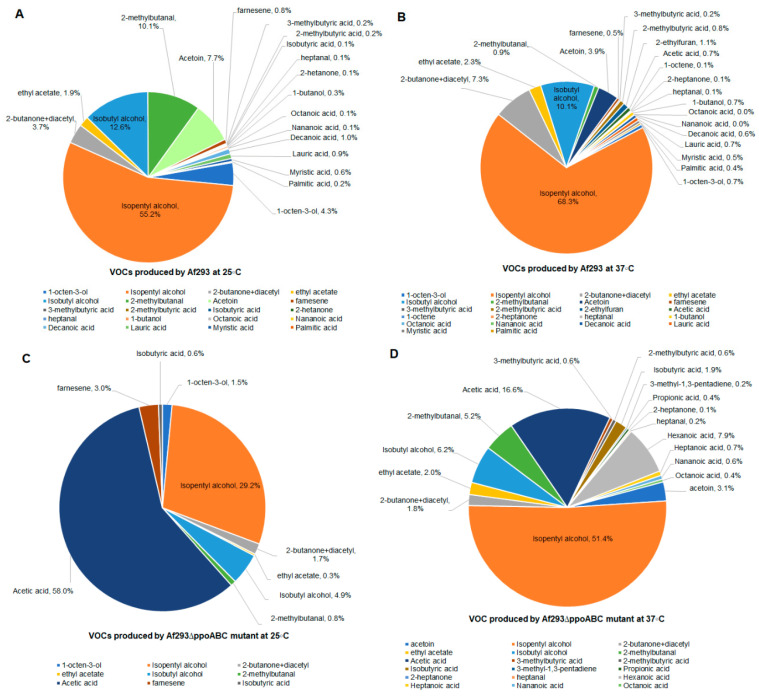
VOCs profiles produced by Af293 and Af293∆*ppoABC* strains. VOCs produced by Af293 pre−grown at 25 °C for 5 days are shown in (**A**); VOCs produced by Af293 pre−grown at 37 °C for 3 days are shown in (**B**); VOCs produced by Af293∆*ppoABC* pre−grown at 25 °C for 5 days are shown in (**C**); and VOCs produced by Af293∆*ppoABC* pre-grown at 37°C for 3 days are shown in (**D**).

Exposure to the VOCs emitted by the wild-type *A. fumigatus* strain was associated with greater delays in metamorphosis and toxicity in *Drosophila* larvae than exposure to VOCs from the triple mutant of this background (Af293∆*ppoABC*). This difference was greater than the difference between wild-type *A. fumigatus* CEA17 and its triple mutant (CEA17 ∆*ppoABC)*. Therefore, we selected Af293 and Af293∆*ppoABC* for VOC profiling using GC–MS analyses. The two strains grown were grown on PDA at 25 °C for 5 days or at 37 °C for 3 days. Compared to the quantity of VOCs emitted by the wild-type Af293, the total concentration of VOCs produced by the blocked oxylipin mutants was only 23.5% at 25 °C, and 45% at 37 °C, showing that the deletion of three COX-like oxygenases significantly decreased the corresponding VOC production. The number of different VOC species detected was also reduced: 20 and 22 were detected from the wild-type Af293 strain at 25 °C and 37 °C, respectively; and 9 and 18 were detected for the Af293∆*ppoABC* strain at 25 °C and 37 °C, respectively (Supplementary Table S2). Thus, the wild-type strain produced more VOCs at higher concentrations compared to its triple mutant. The compositions of VOCs from the wild-type Af293 and the Af293∆*ppoABC* strains at 25 °C and 37 °C are shown in Figure 6, and the amount of each individual VOC emitted by these samples is shown in Supplementary Table S2. The single most abundant VOC produced by the wild-type Af293 strain was isopentyl alcohol (> 55%). Other abundant VOCs included isobutyl alcohol, acetoin, 2-butanone+diacetyl, ethyl acetate, acetoin, 2-methyl butanol and 1-octen-3-ol. More 1-octen-3-ol was produced when the fungus was pre-grown at 25 °C compared to 37 °C. Importantly, 1-octen-3-ol cannot be detected from the Af293∆*ppoABC* mutant pre-grown at 37 °C and was present at only 8% of the level compared to that produced by wild-type Af293. Myristic acid, palmitic acid, lauric acid, 1-butanol, and decanoic acid were detected from the wild-type but not in the deletion mutant (Supplementary Table S2). Compared to the wild-type Af293 strain, the Af293∆*ppoABC* triple mutant produced high concentrations of acetic acid. Moreover, additional acid volatile products not detected in the wild-type *Aspergillus* strain, but detected from the oxylipin mutant, included hexanoic acid, propionic acid, 3-methyl-1,3-pentadiene, and heptanoic acid.

## 4. Discussion

The toxicity of certain fungal VOCs has been studied in rodent models, cultured cell lines and, in a few cases, on human volunteers [24,49,50,51]. In previous work, we developed a *Drosophila* eclosion test and showed that VOCs emitted by living fungal cultures, and by chemical standards of 1-octen-3-ol and other C_8_ volatiles, caused toxicity and death in *Drosophila* larvae and adults [15,16]. These studies have implications for human health because it has been hypothesized that fungal VOCs contribute to the negative health effects experienced by people who live near compost facilities [52] or in water-damaged buildings [25], and contribute to the etiology of a condition variously called “sick building syndrome or “damp building syndrome.” [53,54]. A limited number of controlled studies have shown that VOCs such as 1-octen-3-ol, ethanol and certain air pollutants are harmful to human health [24,55,56,57].

We had two objectives in this work. First, we expanded our earlier work that tested the toxicity of volatiles from *A. fumigatus* in the *Drosophila* model to identify the specific volatiles that interfered with fly metamorphosis and eclosion. As part of this goal, we conducted the fly eclosion test using exposure to VOCs by growing *A. fumigatus* mutants blocked in the oxylipin pathway, and then analyzed the VOCs produced by wild-type *A. fumigatus* and its blocked oxylipin mutant using GC–MS. In addition, because *A. fumigatus* functions as a pathogen at the human body temperature (37 °C), as a subsidiary part of this goal, we compared the toxigenic effects of fungi pre-grown at 37 °C with those pre-grown at 25 °C. *Drosophila* does not grow at 37 °C, so all our exposure studies for the eclosion assay were conducted at the lower temperature. Secondly, we sought to differentiate between the toxigenic effects of VOCs on wild-type fly eclosion and the pathogenic effects of *A. fumigatus*, as studied in *Drosophila* models for aspergillosis. The eclosion test for volatile toxicity involves growing larvae and fungi in a common atmosphere without any physical contact between the two organisms. In contrast, the *Drosophila* models for aspergillosis have utilized Toll-deficient flies and require physical contact between the flies and the fungus [58,59]. Therefore, instead of using wild-type flies in our eclosion assay, we repeated our VOC exposure studies using flies carrying mutations in different innate immune pathway genes. Because the immune-deficient *NOS* and Imd (*Rel^E20^)* fly strains carry the white-eyed genotype, and the *Rel^E20^Spz^4^* strain (blocked Imd and Toll pathway) carries the red-eyed genotype, either white-eyed or red-eyed flies with intact immune systems were used as control strains.

“Oxylipin” is the collective word for all oxygenated lipids. These compounds are produced enzymatically by lipoxygenase (LOX) and cyclooxygenases (COX), as well as non-enzymatically by autoxidative processes associated with reactive oxygen species (ROS). The best understood family of fungal oxylipins are the psi factors (“precocious sexual inducer” factors), which were originally described regarding *Aspergillus nidulans*, where they alter the ratio of asexual to sexual sporulation [60]. The genes that produce psi factors encode three oxygenases and are named PpoA, PpoB and PpoC, and are highly comparable to COX enzymes [21,61]. This group of fungal COX-like oxygenases are now called, generically, Ppo enzymes [35,62,63,64]. In *A*. *fumigatus*, a *ppoABC* RNAi-silenced mutant is hypervirulent in a murine model of pulmonary aspergillosis [44].

Eight carbon oxylipins, including 1-octen-3-ol, 3-octanone, and 3-octanol, are derived from the longer-chain oxylipins as polyunsaturated fatty acids such as arachidonic acid, oleic acid, linoleic, and linolenic acids are broken down [65,66]. The expression of PpoC in *A. nidulans* catalyzes the breakdown of linoleic acid into a wide range of compounds including 1-octen-3-ol, 2-octen-1-ol, 2-octenal, and 3-octanone [67], and a homologue of *ppoC* is necessary for the production of 1-octen-3-ol in *Aspergillus luchuensis* [35]. Many of these volatile oxylipins function in fungal development and reproduction [68]. In our previous study, the two most common and toxic VOCs produced by *A*. *fumigatus* were 1-octen-3-ol and isopentyl alcohol, with 1-octen-3-ol comprising over 60% of total VOCs [17]. In this study, 1-octen-3-ol constituted only 4.3% of the total VOCs produced by the wild-type strain Af293 at 25 °C and only 0.7% at 37 °C. Isopentyl alcohol was the major VOC produced by the wild-type Af293 at both temperatures. In addition to 1-octen-3-ol and isopentyl alcohol, wild-type Af293 produced isobutyl alcohol, acetoin, 2-butanone+diacetyl, ethyl acetate, acetoin, and 2-methyl butanol. The deletion of the *ppoABC* genes from wild-type Af293 caused the absence or significant reduction in several volatiles, including 1-octen-3-ol, isopentyl alcohol, 2-butanone+diacetyl, ethyl acetate, isobutyl alcohol, 2-methylbutanal, and acetoin (Figure 6).

In this study, to determine whether Ppo-derived eight-carbon volatile oxylipins played a role in observed toxicity, we compared the effects of VOCs produced by wild-type *A. fumigatus* and triple mutants (∆*ppoABC*) in the eclosion test using wild-type *Drosophila* larvae. In general, VOCs emitted by the wild-type Af293 strain had more toxic effects on all stages of fruit fly metamorphosis than VOCs from the control CEA17∆*ku80* stain and their derivative triple mutants (Figure 5). Exposure to VOCs emitted by the wild-type Af293 pre-grown at 37 °C had more negative effects on the *Drosophila* flies than exposure to VOCs did when the fungus was pre-grown at 25 °C, i.e., the larvae experienced the longest delays in pupal formation, as well as the lowest eclosion rates (Figure 2B,C). Furthermore, as hypothesized, exposure to the VOCs from the triple mutants (∆*ppoABC*) had far fewer toxic effects on the metamorphosis of all the tested fruit fly strains (Figure 5).

The ability to set off the immune system and repel pathogenic invaders is an essential part of organismal health. The discovery that the Toll pathway is required for protection against fungal infection in *Drosophila* was crucial in studies of both mammalian and *Drosophila* innate immunity [69]. Toll does not directly recognize microbial determinants but is activated by the binding of the ligand *Spätzle*, which occurs after fungal determinants (mainly glucans) are detected by circulating the pattern recognition receptors that induce, by an unknown mechanism, the activation of the proteolytic cascade that leads to the cleavage of Spätzle and subsequent Toll activation [70]. Thus, in *Drosophila* and other insects, the presence of Gram-positive bacteria and fungi are detected indirectly by Spätzle. After the discovery of Toll, a second signaling cascade, the immune deficiency (Imd) pathway, was found by subsequent genetic screens when scientists looked for *Drosophila* mutants that lacked innate responses to infection. Flies carrying Toll mutations are more resistant to physical contact with fungi and Gram-positive bacteria than the Imd mutant flies, while the Imd pathway responds to contact with Gram-negative bacteria [42]. In summary, both Toll and Imd are important in initiating pathogen recognition; flies that mutate for both the Imd and the Toll signaling pathways are highly susceptible to fungal or bacterial infections, and thus are vital for antimicrobial resistance in *Drosophila* [71,72]. Medical mycologists have exploited this fact and employed Toll-deficient *Drosophila* in fly models for studying aspergillosis [58].

In earlier work in our laboratory, adult flies exposed to low concentrations of volatile-phase 1-octen-3-ol in the presence of nitric oxide synthase inhibitors such as L-NAME and minocycline survived longer than flies exposed to 1-octen-3-ol alone, indicating that the toxicity of 1-octen-3-ol is partly mediated via excessive nitric oxide activity. It is known that *Drosophila* nitric oxide synthase (NOS) regulates a number of biological processes, including the host immune response [73,74]. When the genes were overexpressed in the *Drosophila* model, there was an increase in the survival rate by roughly 5 days [75]. Nitric oxide is thought to act upstream of the Imd pathway. In parallel research, paraquat has been used in a fly model of Parkinson’s disease. Paraquat exposure leads to *Drosophila* neurotoxicity and the activation of Relish, indicating a link between exposure to this environmental toxin and the modulation of innate immunity. Knockdown mutants of Relish are resistant to paraquat [76].

In our current work, when flies with a mutation in one of their immune pathways were used in the eclosion assay, the toxic effects of VOCs from *A. fumigatus* were diminished. The absence of toxic effects was particularly striking for the Toll pathway mutants, where the flies carrying the *Spätzle* mutation were similar to the controls in both the timing of metamorphosis and the successful completion of eclosion. Because *Drosophila* Toll plays a key role in recognizing fungal infections [59], it seems counterintuitive that *Spätzle* mutants were more resistant to *Aspergillus* volatiles than flies with a wild-type immune system were.

To date, the overwhelming majority of research on the recognition of pathogens in flies has focused on the physical contact of the fly with the cell wall components and other macromolecular components of microbial pathogens. In mammals, fungal-associated molecular patterns are a subset of pathogen-associated molecular patterns (“PAMPS”), and are known to include fungal cell wall constituents and intracellular components such as DNA [77]. The fact that *Drosophila* flies respond to VOCs shows that much smaller molecules also can be detected at a distance and suggests that the small volatile molecules that are characteristic of the odor compounds emitted by fungi and other microbes can also be identified as “danger signals” by the *Drosophila* immune system. We hypothesize that some fungal volatile molecules may lead to the dysregulation of inflammatory responses and fly toxicity.

We recognize that these data on the effects of volatile compounds on *Drosophila* metamorphosis constitute a preliminary study. We do not know which specific volatile compound or compounds cause the activity leading to toxicity in wild-type flies, nor is the mechanism of *Drosophila* death clear. However, in this study, we have demonstrated that for flies exposed to VOCs from *A*. *fumigatus* strain 293, the possession of a healthy immune system is a liability. *Aspergillus* volatiles may serve as molecular signals that trigger the negative inflammatory process.

In summary, our results demonstrate that VOCs emitted from *A*. *fumigatus* can delay metamorphosis and cause toxicity in wild-type *Drosophila* flies without physical contact between the flies and the fungi. The toxigenic effects of VOCs are less pronounced when the eclosion test uses fly larvae carrying *NOS* and Imd (*Rel^E20^*) mutations and were almost absent in flies carrying the Toll *Spätzle* mutation. Because *Spätzle* is a circulating, cytokine-like endogenous molecule that acts as a ligand to activate Toll receptors and thereby sets off an effective immune response, these data imply that the mortality we have observed in wild-type flies is associated with an overreaction of the fly innate immune system. Most studies that have demonstrated how the immune system response can overreact and cause tissue damage have involved physical contact between the host and the pathogen and/or its macromolecular cellular components. In this current study, only gas phase molecules are in contact with the *Drosophila* larvae, suggesting that flies can perceive fungal pathogens at a distance through their characteristic volatile emissions. We postulate that the *Toll* pathway may be responsible for much of the volatile toxicity observed in the *Drosophila* eclosion test. Finally, these findings may provide new hypotheses for studying the etiology and symptomology of human conditions associated with immune reactions to fungi, such as allergies, asthma, hypersensitivity pneumonitis, and possibly “sick building syndrome”.

## Figures and Tables

**Table 1:** *Aspergillus fumigatus* and *Drosophila melanogaster* strains used in this study.

*A. fumigatus*	Strains	Genotype	References
Af293	Af293	wild type	[37]
CEA17∆*ku80*	CEA10 derivative	*pyrG1,* Δ*ku80::A.fumi.pyrG*	[38]
CEA17 Δ*ku80*∆*argB*∆*ppoA*	TMN19.1	*pyrG1,* ∆*akuB::pyrG, pyrG,* Δ*argB (AFUB_064280)::A.p.pyrG;* Δ*A.p.pyrG;* ∆*ppoA(AFUB_067850)::A.p.pyrG*	this study
Af293 ∆*ppoC*∆*ppoA*	TMN20.11	∆*ppoC::A. nidulans argB; argB1 pyrG1;* ∆*ppoA(Af4g10770)::A.p.pyrG*	this study
CEA17 Δ*ku80*∆*ppoA*∆*ppoC*	TMN28.2	*pyrG1,* ∆*akuB::pyrG, pyrG1,*Δ*argB (AFUB_064280)::A.p.pyrG;* Δ*A.p.pyrG;*∆*ppoA(AFUB_067850)::A.p.pyrG;* ∆*ppoC(AFUB_037060)::A.f. argB*	this study
Af293∆*ppoABC* (Af293∆*ppoA*∆*ppoC*∆*ppoB*)	TMN31.10	Δ*ppoC::A. nidulans argB; argB1pyrG1;*Δ*ppoA(Af4g10770)::A. p. pyrG;* Δ*ppoB (Afu4g00180)::six*	this study
CEA17∆*ku80*∆*ppoABC* (CEA17Δ*ku80*∆*ppoA*∆*ppoC*∆*ppoB*)	TMN32.1	*pyrG1,* Δ*akuB::pyrG, pyrG1,*Δ*argB (AFUB_064280)::A.p.pyrG;* Δ*A.p.pyrG;* Δ*ppoA(AFUB_067850)::A.p.pyrG;* Δ*ppoC(AFUB_037060)::A.fumi. argB;* Δ*ppoB(AFUB_100690)::six*	this study
**D. melanogaster** **BDSC ID** **Type of mutations**	**Genotype**	**Human ortholog**	**References**
6598 white-eye, wild-type *w**1118***	*y1 w1118*		[39]
24283 white-eye, NOS (nitrate oxide synthase) mutant	*w1118;* Mi{ET1}Nos[MB04018]	NOS1 *(Nos)*	[39,40]
9457 white-eyed, *Rel^E20^* gene to induce Imd pathway	*w1118*; Rel[E20]e[s]	NF-kB *(Rel)*	[41]
5 red-eyed, wild-type strain	Oregon-R-C		[42]
10719 red-eyed, *spz^6^* gene to induce Toll pathway	*w1118*; PBac{w[+mC] = PB}spz6[c01763]	TNF-receptor	[41,43]
55718 red-eyed, *Rel^E20^spz^4^* double mutant of Imd and Toll pathways	Rel[E20] spz [4]/TM6C, Sb [1] Tb [1]	TNF-receptor	[42]

## Supplementary Materials

The following supporting information can be downloaded at: https://www.mdpi.com/article/10.3390/jof9040402/s1, Figure S1: Southern analyses of mutant strains in this study. Figure S2: Growth and stress tests of Af293 and CEA17 wild-type strains and the corresponding ΔppoABC mutants.; Table S1: Primers used in this study.; Table S2: VOCs emitted by the wild-type Af293 and its lipoxygenase triple mutant strain pre-grown at 25 °C for 5 days or 37 °C for 3 days by using purge and trap-thermal desorption method.

## References

[1] Latge J.-P., Chamilos G. (2019). Aspergillus fumigatus and aspergillosis in 2019. Clin. Microbiol. Rev..

[2] Latgé J.-P. (1999). Aspergillus fumigatus and aspergillosis. Clin. Microbiol. Rev..

[3] Gregg K.S., Kauffman C.A. (2015). Invasive aspergillosis: Epidemiology, clinical aspects, and treatment. Semin. Respir. Crit. Care Med..

[4] Dagenais T.R., Keller N.P. (2009). Pathogenesis of Aspergillus fumigatus in invasive aspergillosis. Clin. Microbiol. Rev..

[5] Koehler P., Bassetti M., Chakrabarti A., Chen S.C., Colombo A.L., Hoenigl M., Klimko N., Lass-Flörl C., Oladele R.O., Vinh D.C. (2020). Defining and managing COVID-19-associated pulmonary aspergillosis: The 2020 ECMM/ISHAM consensus criteria for research and clinical guidance. Lancet Infect. Dis..

[6] Chamilos G., Lionakis M.S., Lewis R.E., Kontoyiannis D.P. (2007). Role of mini-host models in the study of medically important fungi. Lancet Infect. Dis..

[7] Ben-Ami R., Lamaris G.A., Lewis R.E., Kontoyiannis D.P. (2010). Interstrain variability in the virulence of Aspergillus fumigatus and Aspergillus terreus in a Toll-deficient Drosophila fly model of invasive aspergillosis. Med. Mycol..

[8] Lionakis M.S., Kontoyiannis D.P. (2012). Drosophila melanogaster as a model organism for invasive aspergillosis. Host-Fungus Interactions.

[9] Jahn B., Boukhallouk F., Lotz J., Langfelder K., Wanner G., Brakhage A.A. (2000). Interaction of human phagocytes with pigmentless Aspergillus conidia. Infect. Immun..

[10] Raffa N., Keller N.P. (2019). A call to arms: Mustering secondary metabolites for success and survival of an opportunistic pathogen. PLoS Pathog..

[11] Almaliki H.S., Martinez S., Piszczatowski P., Bennett J.W. (2017). Drosophila melanogaster as a model for studying Aspergillus fumigatus. Mycobiology.

[12] Kowalski C.H., Kerkaert J.D., Liu K.-W., Bond M.C., Hartmann R., Nadell C.D., Stajich J.E., Cramer R.A. (2019). Fungal biofilm morphology impacts hypoxia fitness and disease progression. Nat. Microbiol..

[13] Bhabhra R., Miley M.D., Mylonakis E., Boettner D., Fortwendel J., Panepinto J.C., Postow M., Rhodes J.C., Askew D.S. (2004). Disruption of the Aspergillus fumigatus gene encoding nucleolar protein CgrA impairs thermotolerant growth and reduces virulence. Infect. Immun..

[14] Lionakis M.S., Kontoyiannis D.P. (2010). The growing promise of Toll-deficient Drosophila melanogaster as a model for studying Aspergillus pathogenesis and treatment. Virulence.

[15] Zhao G., Yin G., Inamdar A.A., Luo J., Zhang N., Yang I., Buckley B., Bennett J.W. (2017). Volatile organic compounds emitted by filamentous fungi isolated from flooded homes after Hurricane Sandy show toxicity in a Drosophila bioassay. Indoor Air.

[16] Inamdar A.A., Bennett J.W. (2015). Volatile organic compounds from fungi isolated after Hurricane Katrina induce developmental defects and apoptosis in a Drosophila melanogaster model. Environ. Toxicol..

[17] Almaliki H.S., Angela A., Goraya N.J., Yin G., Bennett J.W. (2021). Volatile organic compounds produced by human pathogenic fungi are toxic to Drosophila melanogaster. Front. Fungal Biol..

[18] Fischer G.J., Bacon W., Yang J., Palmer J.M., Dagenais T., Hammock B.D., Keller N.P. (2017). Lipoxygenase activity accelerates programmed spore germination in Aspergillus fumigatus. Front. Microbiol..

[19] Niu M., Steffan B.N., Fischer G.J., Venkatesh N., Raffa N.L., Wettstein M.A., Bok J.W., Greco C., Zhao C., Berthier E. (2020). Fungal oxylipins direct programmed developmental switches in filamentous fungi. Nat. Commun..

[20] Niu M., Keller N.P. (2019). Co-opting oxylipin signals in microbial disease. Cell. Microbiol..

[21] Tsitsigiannis D.I., Kowieski T.M., Zarnowski R., Keller N.P. (2005). Three putative oxylipin biosynthetic genes integrate sexual and asexual development in Aspergillus nidulans. Microbiology.

[22] Fischer G.J., Keller N.P. (2016). Production of cross-kingdom oxylipins by pathogenic fungi: An update on their role in development and pathogenicity. J. Microbiol..

[23] Davis T.S., Crippen T.L., Hofstetter R.W., Tomberlin J.K. (2013). Microbial volatile emissions as insect semiochemicals. J. Chem. Ecol..

[24] Wålinder R., Ernstgård L., Norbäck D., Wieslander G., Johanson G. (2008). Acute effects of 1-octen-3-ol, a microbial volatile organic compound (MVOC)-An experimental study. Toxicol. Lett..

[25] Douwes J. (2009). Building dampness and its effect on indoor exposure to biological and non-biological pollutants. Who Guidelines for Indoor Air Quality: Dampness and Mould.

[26] Mølhave L. (2009). Volatile organic compounds and sick building syndrome: Chapt 8. Environmental Toxicants, Human Exposures and Their Health Effects.

[27] Bennett J.W., Inamdar A.A. (2015). Are some fungal volatile organic compounds (VOCs) mycotoxins?. Toxins.

[28] Combet E., Henderson J., Eastwood D.C., Burton K.S. (2006). Eight-carbon volatiles in mushrooms and fungi: Properties, analysis, and biosynthesis. Mycoscience.

[29] Korpi A., Jarnberg J., Pasanen A.L. (2009). Microbial volatile organic compounds. Crit. Rev. Toxicol..

[30] Hung R., Lee S., Bennett J.W. (2015). Fungal volatile organic compounds and their role in ecosystems. Appl. Microbiol. Biotechnol..

[31] Pennerman K., Al-Maliki H., Lee S., Bennett J. (2016). Fungal volatile organic compounds (VOCs) and the genus Aspergillus. New and Future Developments in Microbial Biotechnology and Bioengineering.

[32] Inamdar A.A., Morath S., Bennett J.W. (2020). Fungal volatile organic compounds: More than just a funky smell?. Annu. Rev. Microbiol..

[33] Elmassry M.M., Farag M.A., Preissner R., Gohlke B.-O., Piechulla B., Lemfack M.C. (2020). Sixty-one volatiles have phylogenetic signals across bacterial domain and fungal kingdom. Front. Microbiol..

[34] Miyamoto K., Murakami T., Kakumyan P., Keller N.P., Matsui K. (2014). Formation of 1-octen-3-ol from Aspergillus flavus conidia is accelerated after disruption of cells independently of Ppo oxygenases, and is not a main cause of inhibition of germination. PeerJ.

[35] Kataoka R., Watanabe T., Yano S., Mizutani O., Yamada O., Kasumi T., Ogihara J. (2020). Aspergillus luchuensis fatty acid oxygenase ppoC is necessary for 1-octen-3-ol biosynthesis in rice koji. J. Biosci. Bioeng..

[36] Lionakis M.S., Lewis R.E., May G.S., Wiederhold N.P., Albert N.D., Halder G., Kontoyiannis D.P. (2005). Toll-deficient Drosophila flies as a fast, high-throughput model for the study of antifungal drug efficacy against invasive aspergillosis and Aspergillus virulence. J. Infect. Dis..

[37] Rosowski E.E., Raffa N., Knox B.P., Golenberg N., Keller N.P., Huttenlocher A. (2018). Macrophages inhibit Aspergillus fumigatus germination and neutrophil-mediated fungal killing. PLoS Pathog..

[38] da Silva Ferreira M.E., Kress M.R., Savoldi M., Goldman M.H.S., Härtl A., Heinekamp T., Brakhage A.A., Goldman G.H. (2006). The akuBKU80 mutant deficient for nonhomologous end joining is a powerful tool for analyzing pathogenicity in Aspergillus fumigatus. Eukaryot. Cell.

[39] Inamdar A.A., Hossain M.M., Bernstein A.I., Miller G.W., Richardson J.R., Bennett J.W. (2013). Fungal-derived semiochemical 1-octen-3-ol disrupts dopamine packaging and causes neurodegeneration. Proc. Natl. Acad. Sci. USA.

[40] Bellen H.J., Levis R.W., He Y., Carlson J.W., Evans-Holm M., Bae E., Kim J., Metaxakis A., Savakis C., Schulze K.L. (2011). The Drosophila gene disruption project: Progress using transposons with distinctive site specificities. Genetics.

[41] Yamamoto-Hino M., Goto S. (2016). Spätzle-Processing Enzyme-independent activation of the Toll pathway in Drosophila innate immunity. Cell Struct. Funct..

[42] De Gregorio E., Spellman P.T., Tzou P., Rubin G.M., Lemaitre B. (2002). The Toll and Imd pathways are the major regulators of the immune response in Drosophila. EMBO J..

[43] Thibault S.T., Singer M.A., Miyazaki W.Y., Milash B., Dompe N.A., Singh C.M., Buchholz R., Demsky M., Fawcett R., Francis-Lang H.L. (2004). A complementary transposon tool kit for Drosophila melanogaster using P and piggyBac. Nat. Genet..

[44] Tsitsigiannis D.I., Bok J.-W., Andes D., Nielsen K.F., Frisvad J.C., Keller N.P. (2005). Aspergillus cyclooxygenase-like enzymes are associated with prostaglandin production and virulence. Infect. Immun..

[45] Wiemann P., Perevitsky A., Lim F.Y., Shadkchan Y., Knox B.P., Figueora J.A.L., Choera T., Niu M., Steinberger A.J., Wüthrich M. (2017). Aspergillus fumigatus copper export machinery and reactive oxygen intermediate defense counter host copper-mediated oxidative antimicrobial offense. Cell Rep..

[46] Lim F.Y., Sanchez J.F., Wang C.C., Keller N.P. (2012). Toward awakening cryptic secondary metabolite gene clusters in filamentous fungi. Methods in Enzymology.

[47] Szewczyk E., Nayak T., Oakley C.E., Edgerton H., Xiong Y., Taheri-Talesh N., Osmani S.A., Oakley B.R. (2006). Fusion PCR and gene targeting in Aspergillus nidulans. Nat. Protoc..

[48] Hartmann T., Dümig M., Jaber B.M., Szewczyk E., Olbermann P., Morschhäuser J., Krappmann S. (2010). Validation of a self-excising marker in the human pathogen Aspergillus fumigatus by employing the β-rec/six site-specific recombination system. Appl. Environ. Microbiol..

[49] Korpi A., Kasanen J., Pasanen A. (1999). Sensory irritation of microbially produced volatile organic compounds in mice during repeated exposures. Proceedings of the 3rd International Conference: Bioaerosols, Fungi and Mycotoxins: Health Effects, Assessment, Prevention and Control.

[50] Kreja L., Seidel H.-J. (2002). Evaluation of the genotoxic potential of some microbial volatile organic compounds (MVOC) with the comet assay, the micronucleus assay and the HPRT gene mutation assay. Mutat. Res. /Genet. Toxicol. Environ. Mutagen..

[51] Kreja L., Seidel H.-J. (2002). On the cytotoxicity of some microbial volatile organic compounds as studied in the human lung cell line A549. Chemosphere.

[52] Herr C.E., zur Nieden A., Bödeker R.H., Gieler U., Eikmann T.F. (2003). Ranking and frequency of somatic symptoms in residents near composting sites with odor annoyance. Int. J. Hyg. Environ. Health.

[53] Clark N., Ammann H., Brunekreef B., Eggleston P., Fisk W., Fullilove R., Guernsey J., Nevalainen A., Von Essen S. (2004). Damp Indoor Spaces and Health.

[54] Heseltine E., Rosen J.E. (2009). WHO Guidelines for Indoor Air Quality: Dampness and Mould.

[55] Sahlberg B., Gunnbjörnsdottir M., Soon A., Jogi R., Gislason T., Wieslander G., Janson C., Norback D. (2013). Airborne molds and bacteria, microbial volatile organic compounds (MVOC), plasticizers and formaldehyde in dwellings in three North European cities in relation to sick building syndrome (SBS). Sci. Total Environ..

[56] Pantoja L.D.M., do Nascimento R.F., de Araújo Nunes A.B. (2016). Investigation of fungal volatile organic compounds in hospital air. Atmos. Pollut. Res..

[57] Wålinder R., Ernstgård L., Johanson G., Norbäck D., Venge P., Wieslander G. (2005). Acute effects of a fungal volatile compound. Environ. Health Perspect..

[58] Lemaitre B., Nicolas E., Michaut L., Reichhart J.-M., Hoffmann J.A. (1996). The dorsoventral regulatory gene cassette spätzle/Toll/cactus controls the potent antifungal response in Drosophila adults. Cell.

[59] Lemaitre B., Hoffmann J. (2007). The host defense of Drosophila melanogaster. Annu. Rev. Immunol..

[60] Champe S.P., Rao P., Chang A. (1987). An endogenous inducer of sexual development in Aspergillus nidulans. Microbiology.

[61] Tsitsigiannis D.I., Zarnowski R., Keller N.P. (2004). The lipid body protein, PpoA, coordinates sexual and asexual sporulation in Aspergillus nidulans. J. Biol. Chem..

[62] Brown S.H., Scott J.B., Bhaheetharan J., Sharpee W.C., Milde L., Wilson R.A., Keller N.P. (2009). Oxygenase coordination is required for morphological transition and the host–fungus interaction of Aspergillus flavus. Mol. Plant-Microbe Interact..

[63] Shin K.-C., Seo M.-J., Oh D.-K. (2016). Characterization of a novel 8R, 11S-linoleate diol synthase from Penicillium chrysogenum by identification of its enzymatic products. J. Lipid Res..

[64] Wadman M.W., De Vries R.P., Kalkhove S.I., Veldink G.A., Vliegenthart J.F. (2009). Characterization of oxylipins and dioxygenase genes in the asexual fungus Aspergillus niger. BMC Microbiol..

[65] Assaf S., Hadar Y., Dosoretz C.G. (1997). 1-Octen-3-ol and 13-hydroperoxylinoleate are products of distinct pathways in the oxidative breakdown of linoleic acid by Pleurotus pulmonarius. Enzym. Microb. Technol..

[66] Husson F., Bompas D., Kermasha S., Belin J. (2001). Biogeneration of 1-octen-3-ol by lipoxygenase and hydroperoxide lyase activities of Agaricus bisporus. Process Biochem..

[67] Brodhun F., Schneider S., Göbel C., Hornung E., Feussner I. (2010). PpoC from Aspergillus nidulans is a fusion protein with only one active haem. Biochem. J..

[68] Pohl C.H., Kock J.L. (2014). Oxidized fatty acids as inter-kingdom signaling molecules. Molecules.

[69] Medzhitov R., Janeway C. (2000). Innate immunity. N. Engl. J. Med..

[70] El Chamy L., Leclerc V., Caldelari I., Reichhart J.-M. (2008). Sensing of ‘danger signals’ and pathogen-associated molecular patterns defines binary signaling pathways’ upstream’ of Toll. Nat. Immunol..

[71] Brennan C.A., Anderson K.V. (2004). Drosophila: The genetics of innate immune recognition and response. Annu. Rev. Immunol..

[72] Valanne S., Wang J.-H., Rämet M. (2011). The Drosophila toll signaling pathway. J. Immunol..

[73] Mukherjee T., Kim W.S., Mandal L., Banerjee U. (2011). Interaction between Notch and Hif-α in development and survival of Drosophila blood cells. Science.

[74] Stasiv Y., Regulski M., Kuzin B., Tully T., Enikolopov G. (2001). The Drosophila nitric-oxide synthase gene (dNOS) encodes a family of proteins that can modulate NOS activity by acting as dominant negative regulators. J. Biol. Chem..

[75] Inamdar A.A., Bennett J.W. (2014). A common fungal volatile organic compound induces a nitric oxide mediated inflammatory response in Drosophila melanogaster. Sci. Rep..

[76] Maitra U., Scaglione M.N., Chtarbanova S., O’Donnell J.M. (2019). Innate immune responses to paraquat exposure in a Drosophila model of Parkinson’s disease. Sci. Rep..

[77] Salazar F., Brown G.D. (2018). Antifungal innate immunity: A perspective from the last 10 years. J. Innate Immun..

