# Supplementary material for: Mutational Analysis of Aspergillus fumigatus Volatile Oxylipins in a Drosophila Eclosion Assay
Source: J Fungi (Basel). 2023 Mar 24;9(4):402. doi: 10.3390/jof9040402 (PMC10143813; doi:10.3390/jof9040402)
Supplement: Supplementary file 1 [file jof-09-00402-s001.zip › jof-2226262-SM.pdf]

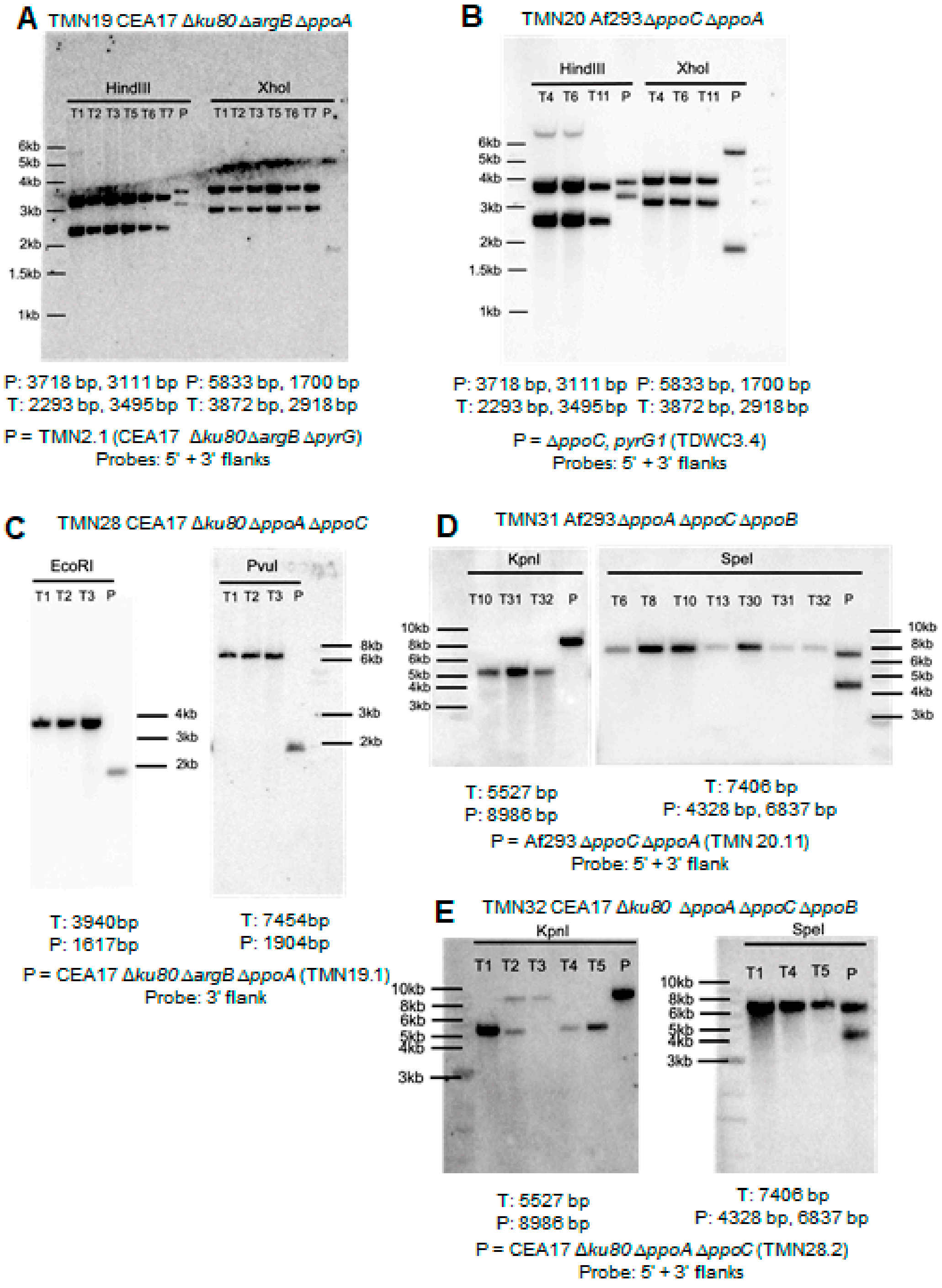

Supplementary Figure S1. Southern analyses of mutant strains in this study. Restriction enzyme digestion, southern blotting and hybridization were performed as mentioned in the Materials and Methods. Single, double and tripled ppo mutants were A. TMN 19 (CEA17 Δku80 ΔargB ΔppoA); B. TMN20 Af293 ΔppoC ΔppoA; C. TMN28 CEA17 Δku80 ΔppoA ΔppoC; D. TMN31 Af293 ΔppoA ΔppoC ΔppoB; E. TMN32 CEA17 Δku80 ΔppoA ΔppoC ΔppoB. Selective transformants were used as parental strains. P = parental strain; T = transformants; probes indicate the DNA fragment used for hybridization.

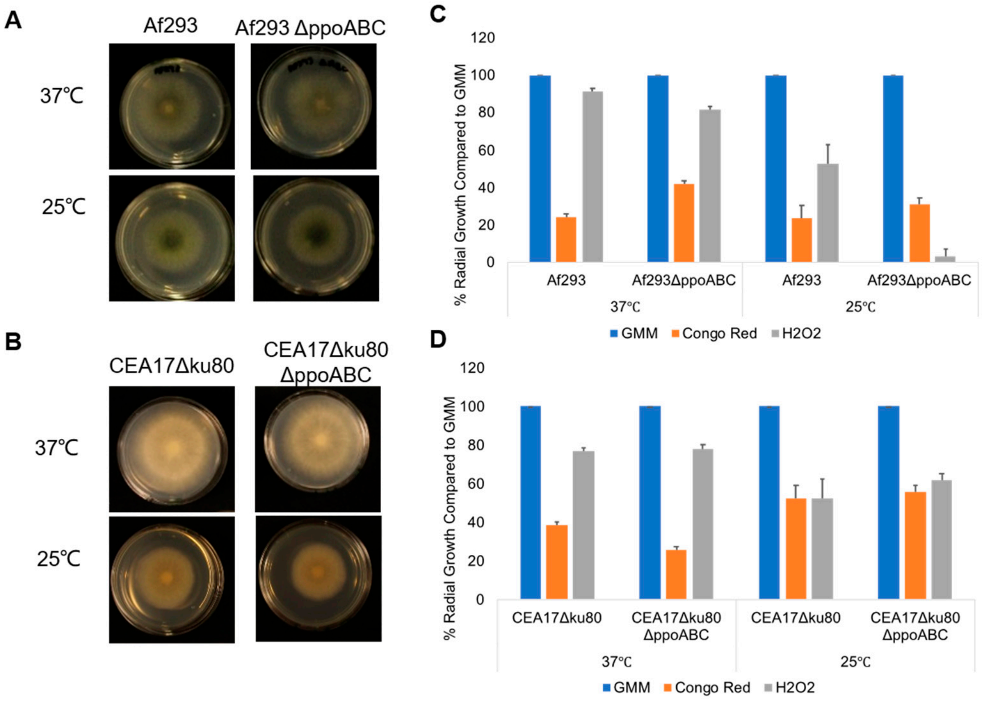

Supplementary Figure S2. Growth and stress tests of Af293 and CEA17 wild-type strains and the corresponding ΔppoABC mutants. A. Strains were cultured on solid GMM plates for 3 and 5 days at 37℃ and 25℃, respectively; B. Strains were cultured on GMM, GMM + Congo Red, or GMM + H_2O_2 at specified temperature. Stressor concentrations used were: 50 μg/mL for Congo Red for both strain backgrounds, 2 mM H_2O_2 for Af293 strains, and 3 mM H_2O_2 for CEA17 strains to achieve comparable levels of inhibition in the wildtypes. Growth diameter of each strain growing under stressors was normalized to their growth diameter of the same strain on GMM. Values represent mean ± SD from three biological replicates.

Supplementary Table S1. Primers used in this study.

Primers	Sequence (5´→3´)	Purpose
GF ppoA del Cassette F	CGCGCGTAATACGACTCACTATAGG	*ppoA* deletion cassette
GF ppoA del Cassette R	AACAAAAGCTGGAGCTCCACC	*ppoA* deletion cassette
MN KOppoA 5´ flank F	GGCGTCGATTTGCTTATCTAAGC	*ppoA* deletion 5´ flank forward for southern analysis
MN KOppoA 5´ flank R	CGACAGCAACTCATGAGGAGG	*ppoA* deletion 5´ flank reverse for southern analysis
MN KOppoA 3´ flank F	GTGACTCGACTGTATGGAGTAG	*ppoA* deletion 3´ flank forward for southern analysis
MN KOppoA 3´ flank R	GATCCAATACATCCACCATGTGC	*ppoA* deletion 3´ flank reverse for southern analysis
MN ppoA ORF F	TCTTCACGGAGTCAGAGTTGTACC	PCR screen of *ppoA* forward
MN ppoA ORF R	CTTTACTTCTGGCAAATCGCCATCC	PCR screen of *ppoA* reverse
MN KOppoC 5´ flank F	GTCAGTGCGCCATAGATAATGC	*ppoC* deletion 5´ flank forward

MN KOppoC 5´ flank R	GAAAATTTGTCTTGGATGCAGACCGCGTTCAATTGTGTACGGATGGAATC	*ppoC* deletion 5´ flank reverse
MN pJMP4 argB F	GAACGCGGTCTGCATCC	*argB* amplification from pJMP4 forward
MN pJMP4 argB R	GGAGAGACCCATACATCCATTTG	*argB* amplification from pJMP4 reverse
MN KOppoC 3´ flank F	ATAGATCAAATGGATGTATGGGTCTCTCCGCGATTCCCAGCTTTGTCTTG	*ppoC* deletion 3´ flank forward
MN KOppoC 3´ flank R	GCATGAAACATAGCAAACGCG	*ppoC* deletion 3´ flank reverse
MN pJMP4 argB 3´ screen F	GCACCTCCTCACCTACAG	*ppoC* deletion 3´ flank screen forward
MN pJMP4 argB 3´ screen R	CCATATTCTCCACCTCAACTGC	*ppoC* deletion 3´ flank screen reverse
MN ppoC ORF F	GTGATCTTTGCCGCAAATGTC	PCR screen of *ppoC* forward
MN ppoC ORF R	TTAGGCATCGTACTGGACACG	PCR screen of *ppoC* reverse
MN KOppoB 5´ flank F	TGGTGCCTCGTGACAAGTC	*ppoB* deletion 5´ flank forward
MN KOppoB 5´ flank R	CTCTATTGACCTATAGGACCTGAGTGATGCCCAAATGACTGGAGCAACCC	*ppoB* deletion 5´ flank reverse
MN KOppoB 3´ flank F	TTAAGTTGAGCATAATATGGTCCATCTAGTGCGGGTGCATTACGTACAAG	*ppoB* deletion 3´ flank forward
MN KOppoB 3´ flank R	GCTGAACTGGACTGCCTC	*ppoB* deletion 3´ flank reverse
MN ppoB ORF F	GCAATGGCAGTGATCTTCACAGC	PCR screen of *ppoB* forward
MN ppoB ORF R	AATGTCCCGGCACATTACATACG	PCR screen of *ppoB* reverse

Supplementary Table S2. VOCs emitted by the wild-type Af293 and its lipoxygenase triple mutant strain pre-grown at 25◦C for 5 days or 37◦C for 3 days by using purge and trap-thermal desorption method.

**VOCs**	**Af293**		**Af293ΔppoABC**	
	25◦C (ng/trap)	37 ◦C (ng/trap)	25 ◦C (ng/trap)	37 ◦C (ng/trap)
1-Octen-3-ol	42.8	8.4	3.5	nd
Isopentyl alcohol	547.2	875.6	67.9	296.3
2-butanone+diacetyl	37.0	93.7	3.9	10.3
ethyl acetate	18.7	29.1	0.7	11.4
Isobutyl alcohol	124.9	129.6	11.5	35.6
2-methylbutanal	99.7	11.2	1.9	29.8
Acetoin	76.5	50.3	nd	17.9
farnesene	7.9	6.5	7.1	nd
3-methylbutyric acid	1.6	3.2	nd	3.2
2-methylbutyric acid	1.5	10.8	nd	3.4
Isobutyric acid	1.1	nd	1.4	11.0
2-hetanone	0.5	nd	nd	nd
heptanal	0.5	0.9	nd	1.3
1-butanol	3.2	8.6	nd	nd
Octanoic acid	1.4	0.6	nd	2.6
Nananoic acid	1.0	0.5	nd	3.7
Decanoic acid	9.8	7.7	nd	nd
Lauric acid	9.0	8.4	nd	nd
Myristic acid	5.5	6.9	nd	nd
Palmitic acid	2.1	4.6	nd	nd
2-ethylfuran	nd	13.6	nd	nd
Acetic acid	nd	9.4	134.9	95.9
1-octene	nd	1.4	nd	nd

2-heptanone	nd	1.7	nd	0.7
Heptanoic acid	nd	nd	nd	4.2
3-methyl-1,3-pentadiene	nd	nd	nd	1.4
Propionic acid	nd	nd	nd	2.3
Hexanoic acid	nd	nd	nd	45.6
Total amount (ng)	991.9	1282.7	232.8	576.6
Total VOCs species	20	22	9	18

Note: the “nd” indicates not detected. Data are the mean values of two individual tests.
